# Summary of the mechanism of ferroptosis regulated by m6A modification in cancer progression

**DOI:** 10.3389/fcell.2025.1507171

**Published:** 2025-04-09

**Authors:** Bin Fan, Gangxian Chen, Shuyi Huang, Ying Li, Zia Ul Haq Nabil, Zuozhang Yang

**Affiliations:** Bone and Soft Tissue Tumors Research Centre of Yunnan Province, Department of Orthopaedics, The Third Affiliated Hospital of Kunming Medical University (Yunnan Cancer Hospital), Kunming, Yunnan, China

**Keywords:** N6-methyladenosine, m6A methyltransferases, m6A demethylase, m6A reading protein, ferroptosis, cancer

## Abstract

The most common form of internal RNA modification in eukaryotes is called n6-methyladenosine (m6A) methylation. It has become more and more well-known as a research issue in recent years since it alters RNA metabolism and is involved in numerous biological processes. Currently, m6A alteration offers new opportunities in clinical applications and is intimately linked to carcinogenesis. Ferroptosis—a form of iron-dependent, lipid peroxidation-induced regulated cell death—was discovered. In the development of cancer, it has become an important factor. According to newly available data, ferroptosis regulates tumor growth, and cancer exhibits aberrant m6A levels in crucial ferroptosis regulatory components. On the other hand, m6A has multiple roles in the development of tumors, and the relationship between m6A-modified ferroptosis and malignancies is quite intricate. In this review, we first give a thorough review of the regulatory and functional roles of m6A methylation, focusing on the molecular processes of m6A through the regulation of ferroptosis in human cancer progression and metastasis, which are strongly associated to cancer initiation, progression, and drug resistance. Therefore, it is crucial to clarify the relationship between m6A-mediated regulation of ferroptosis in cancer progression, providing a new strategy for cancer treatment with substantial clinical implications.

## 1 Introduction

N6-methyladenosine (m6A), the most common post-transcriptional modification of RNA in eukaryotic messenger RNA (mRNA), represents one of over 160 chemical modifications identified in RNA molecules to date (Boccaletto et al., 2022). This modification influences various aspects of RNA metabolism, including translation, splicing, RNA-protein interactions, and RNA stability. The regulation of m6A involves three classes of proteins: writers, readers, and erasers. Writers, comprising an enzyme complex with methyltransferase activity, catalyze the addition of the m6A modification at specific sites, typically located near stop codons and in the 3′-untranslated regions (3′-UTRs). Readers, or m6A-binding proteins, recognize and bind to these modified sites, modulating the biological functions of the RNA. Conversely, erasers, or demethylases, remove the m6A modification, highlighting the dynamic and reversible nature of this process (An and Duan, 2022; Boulias and Greer, 2023). Importantly, m6A is the most prevalent internal RNA modification and plays diverse roles in cancer. These roles can vary significantly depending on the specific location of the m6A motif and context-dependent factors, such as the tumor microenvironment.

Ferroptosis, a distinct form of programmed cell death, was first identified in 2012 and is characterized by the accumulation of iron-dependent lipid peroxides. A central molecular mechanism underlying ferroptosis involves maintaining a delicate balance between antioxidant defense and oxidative damage (Dixon et al., 2012). Ferrous iron or lipoxygenases catalyze the lipid peroxidation of polyunsaturated fatty acid-containing phospholipids (PUFA-PLs), which are abundantly expressed in cell membranes, ultimately leading to cell death. Glutathione peroxidase 4 (GPX4), a key enzyme in the antioxidant defense system, plays an essential role in preventing ferroptosis by neutralizing lipoxygenase-mediated lipid peroxidation and reducing phospholipid hydroperoxides (Yang et al., 2016; Stockwell et al., 2017; Doll et al., 2017; Yang et al., 2014). Ferroptosis has been strongly implicated in the progression of various diseases, including cancer, liver disorders, and neurological conditions (Ryan et al., 2023; Conche et al., 2023; Zheng et al., 2023).

N6-methyladenosine (m6A) methylation, the most prevalent epigenetic modification of messenger RNAs (mRNAs) and non-coding RNAs (ncRNAs), significantly influences RNA metabolism and, consequently, post-transcriptional regulation of protein expression. Ferroptosis itself involves intricate transcriptional changes and post-translational protein modifications that contribute to cell death (Stockwell, 2022). Emerging evidence indicates that m6A plays a regulatory role in ferroptosis by modulating gene expression, thereby affecting tumor growth. For instance, aberrant m6A methylation levels have been observed in key ferroptosis regulators across several tumor types. However, the precise interplay between m6A-modified ferroptosis and cancer progression remains incompletely understood.

This review focuses on the role of m6A methylation in human cancer development and metastasis, with a particular emphasis on the molecular mechanisms through which m6A regulates ferroptosis. Understanding how m6A controls ferroptosis in the context of cancer progression is critical, as it provides novel insights into cancer treatment and holds significant potential for clinical applications.

## 2 M6A regulators

**FIGURE 1:**
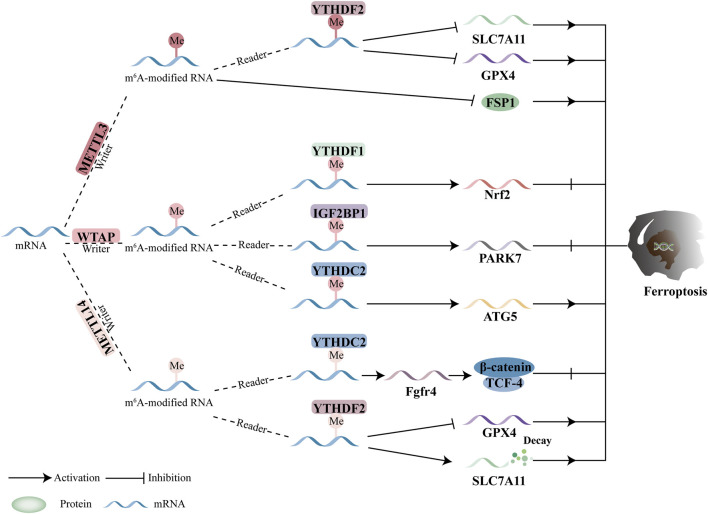
M6A regulators. Writers enter m6A, erasers remove it, and readers recognize it.

m6A regulators are a class of enzymes responsible for catalyzing the m6A modification of RNA molecules, which is the most prevalent post-transcriptional modification in eukaryotic RNA. This modification is essential for various RNA functions, including transcription, splicing, translation, and stability. The initiation, recognition, and removal of m6A modifications are carried out by three categories of regulators: writers, readers, and erasers (Figure 1).

The m6A writers, including METTL3, METTL14, METTL16, WTAP, RBM15/15B, VIRMA, and ZC3H13, primarily form a methyltransferase complex (MTC) between METTL3 and METTL14. This complex is responsible for installing m6A modifications on RNA. METTL14 stabilizes the conformation of the MTC and recognizes specific RNA sequences, while METTL3 catalyzes the transfer of methyl groups from S-adenosylmethionine (SAM) to adenine residues within RNA. The efficiency and specificity of this complex are further enhanced by additional components (Wang et al., 2016; Zeng et al., 2020; Shi et al., 2022). For example, RBM15/15B, an RNA-binding protein, directs the METTL3-METTL14 heterodimer to specific RNA regions (Ping et al., 2014; Patil et al., 2016). VIRMA, also known as vir like m6A methyltransferase associated, interacts with polyadenylation cleavage factors CPSF5 and CPSF6 to recruit the MTC (Yue et al., 2018). Additionally, ZC3H13, a zinc-finger protein, stabilizes the MTC through its interaction with the Wilms Tumor 1-Associated Protein (WTAP) (Chelmicki et al., 2021). METTL16, a methyltransferase-like protein, preferentially catalyzes m6A modification on the U6 small nuclear RNA (snRNA), exhibiting distinct sequence and structural context preferences compared to the MTC (Mendel et al., 2021).

In addition to the MTC, newly identified independent RNA methyltransferases such as METTL5 and METTL16 also catalyze m6A modifications via their methyltransferase domains. On the other hand, m6A erasers, such as obesity-associated protein (FTO) and ALKBH5, function to demethylate m6A-modified bases (Li Y. et al., 2022; Qu et al., 2022). A recently discovered demethylase, ALKBH3, reverses m6A modifications and facilitates RNA demethylation using a similar mechanism (Sun Y. et al., 2023).

Various readers, including members of the YTH domain family, insulin-like growth factor 2 mRNA-binding proteins (IGF2BPs), the heterogeneous nuclear ribonucleoprotein (HNRNP) family, and eukaryotic translation initiation factor 3 (eIF3), detect m6A modifications in response to external stimuli. These interactions directly influence downstream mRNA translation, stability, and decay.

YTH domain-containing protein 1 (YTHDC1) promotes RNA processing and export (Qiao et al., 2023), while YTH domain-containing protein 2 (YTHDC2) reduces RNA abundance to enhance the translation initiation rate of its target mRNA (Li L. et al., 2022). YTH domain family protein 2 (YTHDF2) accelerates mRNA degradation, in contrast to YTH domain family protein 1 (YTHDF1), which acts as a translation initiation factor. In collaboration with these proteins, YTH domain family protein 3 (YTHDF3) functions as an accessory protein in the regulation of target mRNAs (Zaccara and Jaffrey, 2020).

In addition to these, the IGF2BP family (IGF2BP1/2/3) stabilizes mRNA and stimulates translation by interacting with eIF3 (Huang et al., 2020). The heterogeneous nuclear ribonucleoprotein A2/B1 (HNRNPA2/B1) plays a role in the processing of pri-miRNA (Alarcón et al., 2015a). Furthermore, HNRNPC/G binds to m6A-modified mRNAs, influencing their splicing and enrichment, thus contributing to the “m6A switch” phenomenon (Liu et al., 2015; Zhou et al., 2019).

## 3 M6A modification in RNA metabolism

m6A methylation influences various components of RNA metabolism, including the m6A writer complex, readers, erasers, and other regulatory factors, thereby impacting mRNA processing, splicing, translation, and decay. This modification plays a pivotal role in determining the fate and function of RNA molecules (Boulias and Greer, 2023). While the significance of m6A as a post-transcriptional modification has been well established, much remains to be understood regarding the development, dynamics, and regulation of specific m6A sites. Notably, the location of m6A modification sites on RNA can result in distinct functional consequences, leading to complex patterns of mRNA regulation.

According to Berulava et al., m6A modification sites are commonly found within coding sequences (CDS), 5′- or 3′-untranslated regions (UTRs), and translation termination sites. Transcripts with m6A modifications in the 3′-UTR are primarily associated with metabolic and protein-related pathways, including “acetyl-CoA or glycerol biosynthesis” and “positive regulation of protein dephosphorylation.” In contrast, m6A modifications in the 5′-UTR and/or CDS are more frequently linked to intracellular signaling pathways, mitochondrial function, and energy metabolism (Berulava et al., 2020).

An increasing body of research indicates that the processing of non-coding RNAs (ncRNAs) is significantly influenced by m6A modifications. The effects of m6A on microRNAs (miRNAs) can be mediated by a group of m6A regulatory proteins (Qi et al., 2023). For example, METTL3, a key component of the methyltransferase complex (MTC), can modulate the steady-state levels of several miRNAs, including let-7e, miR-1914, miR-128, miR-1246, miR-221/222, and miR-328, thereby promoting miRNA biogenesis (Alarcón et al., 2015b). Similarly, METTL14, another m6A writer enzyme, regulates the production of miRNAs by influencing m6A modification patterns (Fan et al., 2022).

In addition to these MTC components, m6A reader proteins, particularly HNRNPA2B1, are involved in the synthesis and maturation of miRNAs. For instance, depletion of HNRNPA2B1 reduces the processing of the primary miRNA-106b, which in turn promotes the proliferation of non-small cell lung cancer (NSCLC) cells (Chen Z. et al., 2020). Furthermore, prior studies have highlighted the role of m6A reader proteins in regulating circular RNAs (circRNAs). For example, the IGF2BP2 reader protein binds to m6A-modified circQSOX1, regulating miR-326 and miR-330-5p, which subsequently enhances PGAM1 expression and promotes colorectal cancer (CRC) carcinogenesis (Liu Z. et al., 2022).

Moreover, m6A modifications actively participate in regulating various aspects of gene expression and cellular biology, particularly through interactions between long non-coding RNAs (lncRNAs) and miRNAs (Wang et al., 2022). The relationship between ncRNAs and mRNAs is also profoundly influenced by m6A modifications. For instance, the lncRNA lncMIR100HG enhances TCF7L2 mRNA stability through its interaction with HNRNPA2B1, promoting CRC progression (Liu H. et al., 2022). Similarly, hypoxia-induced upregulation of hypoxia-inducible factor-1α (HIF-1α) transcription increases the expression of the lncRNA STEAP3-AS1, which competes with YTHDF2 to stabilize STEAP3 mRNA and protein expression, further facilitating CRC advancement (Zhou L. et al., 2022).

Thus, m6A modifications contribute to miRNA maturation, circRNA translation, and RNA-RNA interactions, in addition to their well-known roles in mRNA processing and function. Overall, many researchers recognize that highly m6A-modified RNAs exhibit diverse biological functions, and m6A regulatory proteins may have significant effects on both ncRNAs and mRNAs. However, further investigation is required to fully elucidate the precise mechanisms of m6A deposition on mRNAs and the functional roles of m6A regulatory proteins within the ncRNA transcriptome.

## 4 The contradictory role of m6A-modified RNA in mediating cancer development

**FIGURE 2:**
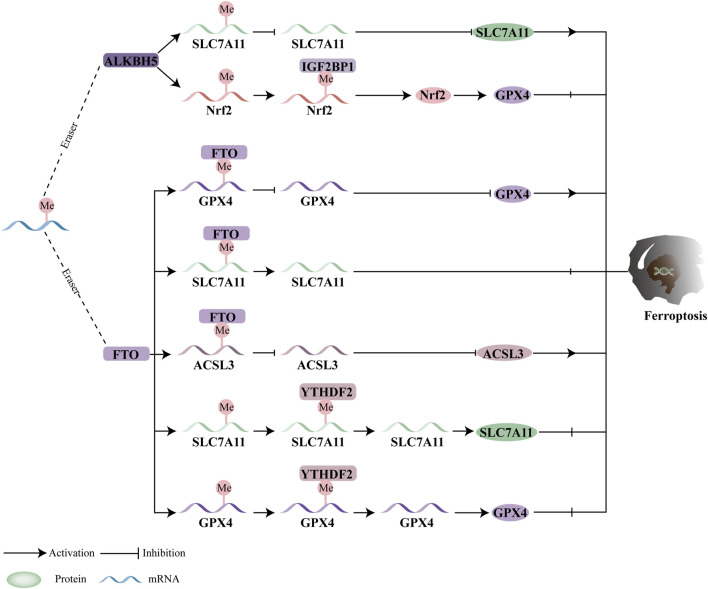
The complex function of m6A alteration in many cancer types.

m6A modification is involved in a wide range of biological processes, both physiological and pathological, with its functions likely determined by the specific location of the m6A motif. The roles of various m6A-related enzymes in cancer are complex and multifaceted, influenced by numerous factors. These enzymes can either promote or inhibit cancer initiation and progression within the same type of tumor. Divergent outcomes are even observed for the same m6A-related enzyme across different cancers (Figure 2). However, the precise mechanisms by which m6A modifications regulate gene expression and contribute to tumorigenesis remain unclear. This section summarizes the latest findings on the intricate roles of m6A-related enzymes, which may have overlapping or identical functions, in the context of cancer.

### 4.1 The complex roles of different m6A-related enzymes in cancer

It has been demonstrated that m6A methylation plays a crucial role in several types of cancer, including head and neck cancer (Jin et al., 2022), nasopharyngeal carcinoma (NPC) (Zhao et al., 2022), breast cancer (BC) (Peng et al., 2021), lung cancer (Fang et al., 2023), gastric cancer (GC) (Sun L. et al., 2023), pancreatic cancer (Li et al., 2023a), hepatocellular carcinoma (HCC) (Hu et al., 2023), colorectal cancer (CRC) (Zeng et al., 2023), bladder cancer (Ni et al., 2022), endometrial cancer (Zhang L. et al., 2021), acute myeloid leukemia (AML) (Yankova et al., 2021), and glioblastoma (GBM) (Fang et al., 2021), among others. However, m6A exhibits a dynamic and paradoxical role across different cancers.

Yu et al. found that WTAP enhances the stability of HK2 mRNA by binding to the 3′-UTR m6A site, thereby accelerating the Warburg effect in gastric cancer (GC) and promoting its progression (Yu et al., 2021). Another study demonstrated that WTAP expression is significantly elevated in nasopharyngeal carcinoma (NPC), where it is associated with poor prognosis. WTAP facilitates NPC development and metastasis by stabilizing the lncRNA DIAPH1-AS1, a mechanism that is also dependent on the m6A reader IGF2BP2 (Li ZX. et al., 2022).

METTL3, a key m6A methyltransferase, has been shown to decrease the expression of the tumor suppressor gene APC, exerting carcinogenic effects. METTL3 is highly expressed in esophageal squamous cell carcinoma (ESCC) and is linked to poor prognosis in patients (Wang et al., 2021). Moreover, METTL3 is associated with poor prognosis in non-small cell lung cancer (NSCLC). Knockdown of METTL3 significantly suppresses tumor development *in vivo* and inhibits NSCLC cell migration and invasion (Chen M. et al., 2023). In lung adenocarcinoma (LUAD), METTL3 triggers the oncogenic molecule c-MYC by enhancing the stability of lncRNA LCAT3, interacting with the upstream protein FUBP1, and promoting tumor cell growth, infiltration, and dissemination (Qian et al., 2021).

In pancreatic cancer cells, METTL14 directly targets the downstream mRNA of PERP, increasing its turnover and decreasing both mRNA and protein levels of PERP, which promotes cancer cell proliferation and metastasis (Wang et al., 2020). Studies also indicate that METTL16 is associated with poor prognosis in colorectal cancer (CRC), as it binds with IGF2BP1 to significantly increase SOGA1 expression and mRNA stability, contributing to METTL16-mediated glycolysis and CRC growth (Wei et al., 2023).

Methyltransferases have also been implicated in the development of drug resistance. For example, METTL3 stimulates the growth, invasion, and migration of pancreatic cancer cells both *in vitro* and *in vivo*. METTL3 modifies DDX23 mRNA in a YTHDF1-dependent manner, promoting the progression of pancreatic ductal adenocarcinoma (PDAC) and gemcitabine resistance (Lin C. et al., 2023). Similarly, LUAD gefitinib resistance may be enhanced by METTL3-induced lncRNA SNHG17 through epigenetic suppression of LATS2 expression (Zhang H. et al., 2022). METTL3 has also been identified as a potential target for CRC immunotherapy. Furthermore, m6A modification plays a critical role in immune modulation. In a m6A-dependent manner, METTL3 upregulates BHLHE41 expression, which in turn triggers CXCL1 transcription and enhances the migration of myeloid-derived suppressor cells (MDSCs) *in vitro* (Chen et al., 2022).

FTO, which is often downregulated in malignancies, plays a critical role in cancer progression. Silencing of FTO has been shown to enhance cell motility, invasion, and tumor growth across various epithelial cancers. Downregulation of FTO demethylase promotes epithelial-mesenchymal transition (EMT)-mediated epithelial tumor progression and increases sensitivity to Wnt inhibitors (Jeschke et al., 2021). Furthermore, studies have revealed a negative correlation between reduced FTO expression and poor survival in patients with lung cancer. When FTO is downregulated, m6A levels on MYC mRNA rise, which in turn attracts YTHDF1 binding. This binding enhances MYC mRNA translation, leading to increased glycolysis and tumorigenesis in tumor cells (Yang et al., 2021).

Conversely, elevated FTO expression in bladder cancer tissues is associated with improved clinical outcomes and acts as an oncogene, promoting the viability and tumorigenicity of bladder cancer cells. Mechanistically, FTO modifies m6A RNA to regulate the MALAT1/miR-384/MAL2 axis, accelerating bladder cancer development (Tao et al., 2021). Similarly, ALKBH5 expression in hepatocellular carcinoma (HCC) is linked to poor prognosis. Through the ALKBH5/MAP3K8 axis, ALKBH5 promotes proliferation, metastasis, and the recruitment of PD-L1+ macrophages in HCC (You et al., 2022).

To further investigate the significance of m6A in various diseases, it is essential to explore the role of methylation readers in tumor development. High expression levels of YTHDF1 are associated with more aggressive tumor progression and poorer overall survival. Inhibition of YTHDF1 can reduce tumor incidence and suppress gastric cancer (GC) cell proliferation both *in vitro* and *in vivo*. In an m6A-dependent manner, YTHDF1 promotes the translation of the crucial Wnt receptor frizzled7 (FZD7), and mutant YTHDF1 increases FZD7 expression. This leads to overactivation of the Wnt/β-catenin pathway, further promoting GC development (Pi et al., 2021).

Additionally, YTHDC1 is overexpressed and exhibits oncogenic functions in acute myeloid leukemia (AML) (Sheng et al., 2021). Overexpression of IGF2BP2 in pancreatic cancer patients is associated with poor prognosis. Together, IGF2BP2 and DANCR enhance the cancerous properties of pancreatic cancer, contributing to its pathogenesis. Inhibition of IGF2BP2 can regulate lncDANCR, reduce cell proliferation, and stabilize DANCR RNA through m6A modification (Hu et al., 2020).

In summary, m6A plays a pivotal role in various cancers. However, the regulation of m6A levels in tumors is bidirectional, exhibiting both pro- and anti-cancer effects. The precise mechanisms underlying these effects warrant further investigation.

### 4.2 Contradictory roles of methylation-related enzymes with opposite effects in the same tumor

Contradictory expression patterns of m6A-related enzymes can occur within tumors, with methylation-related enzymes exhibiting opposing effects. Furthermore, different m6A-related enzymes may have conflicting functions within the same tumor. For example, in gastric cancer (GC), silencing of ALKBH5 enhances tumor invasion and metastasis, whereas low ALKBH5 expression is associated with distant metastases and lymph node involvement in clinical settings (Hu et al., 2022). In contrast, the expression of METTL14 in GC follows an opposite trend; overexpression of METTL14 inhibits GC progression through the methylation modification of circORC5 (Fan et al., 2022).

ALKBH5 is frequently overexpressed in acute myeloid leukemia (AML), which correlates with poor prognosis in AML patients. ALKBH5 promotes malignancy in AML by post-transcriptionally regulating key targets, such as TACC3, contributing to tumorigenesis (Shen et al., 2020). Additionally, in t (Yang et al., 2014; Li Y. et al., 2022) AML, ALKBH5 demethylates ITPA mRNA, enhancing its stability and expression, thereby promoting leukemia development (Li et al., 2023b). In contrast, METTL3, as a methyltransferase, plays a pro-cancer role in AML by increasing the half-life of ITGA4 mRNA through m6A methylation, thereby elevating ITGA4 protein levels and facilitating chemoresistance through enhanced AML cell homing and engraftment (Li M. et al., 2022). Treatment with STM2457, a selective catalytic inhibitor of METTL3, reduces tumor growth while promoting differentiation and apoptosis in AML (Yankova et al., 2021).

In pancreatic cancer, ALKBH5 has been shown to stimulate tumor growth. Conversely, WISP2 antisense RNA 1 (KCNK15-AS1) and potassium two-pore domain channel subfamily K member 15 (KCNK15) inhibit the migration and invasion of pancreatic cancer cells. ALKBH5 overexpression downregulates KCNK15-AS1, accelerating tumor growth (He et al., 2021). In contrast, METTL3 contributes to pancreatic cancer development by promoting SMS mRNA degradation via the METTL3-IGF2BP3 axis in an m6A-dependent manner (Guo et al., 2022).

### 4.3 m6A enzymes of the same or similar action show contradictory effects in different tumors

m6A enzymes with similar functions in different tumors can exhibit contradictory roles. For instance, in gastric cancer (GC), METTL3, a major RNA N6-adenosine methyltransferase, is upregulated. Clinically, elevated METTL3 levels serve as an indicator of poor prognosis. METTL3 is also essential for epithelial-mesenchymal transition (EMT) processes *in vitro* and metastasis *in vivo* (Yue et al., 2019). Furthermore, clinical studies have shown a strong correlation between the cytoplasmic localization of METTL3 and GC progression, suggesting that METTL3’s subcellular distribution may serve as a prognostic marker for cancer patients (Wei et al., 2022). In contrast, METTL14, another m6A writer, is downregulated in colorectal cancer (CRC), and its decreased expression promotes tumor spread. Knockdown of METTL14 results in the elimination of m6A modifications on SOX4 mRNA, which in turn increases SOX4 mRNA expression. This process is mediated through the YTHDF2-dependent pathway, which is crucial for METTL14-induced degradation of SOX4 mRNA (Chen X. et al., 2020).

In pancreatic cancer (PC), ALKBH5 knockdown accelerates tumor development, while its overexpression enhances tumor growth *in vivo* but inhibits migration, invasion, and proliferation of pancreatic cancer cells *in vitro* (Guo et al., 2020). Conversely, in esophageal squamous cell carcinoma (ESCC), overexpression of FTO promotes LINC00022-dependent cell proliferation and tumor growth (Cui et al., 2021).

Similar contradictions are seen with m6A reader proteins. For example, the overexpression of YTHDF3 in liver cancer is associated with poor prognosis, and knockdown of YTHDF3 inhibits lung metastasis and HCC cell proliferation (Zhou R. et al., 2022). On the other hand, reduced expression of YTHDC2 correlates with worse clinical outcomes in lung adenocarcinoma (LUAD). YTHDC2 suppresses LUAD growth by destabilizing SLC7A11 mRNA in an m6A-dependent manner (Ma et al., 2021a).

Conflicting roles are also observed for the same m6A enzyme across different tumors. For instance, METTL14 is downregulated in gastric cancer (GC) tissues, where its low expression is associated with a poorer prognosis. Knockdown of METTL14 in GC cells facilitates their proliferation and invasion (Fan et al., 2022). In contrast, METTL14 is highly expressed in acute myeloid leukemia (AML) cells, where silencing it induces terminal myeloid differentiation in both AML and normal hematopoietic stem and progenitor cells (HSPCs), thereby preventing AML cell survival and proliferation (Weng et al., 2018).

Similarly, high levels of the m6A demethylase FTO correlate with poor prognosis in nasopharyngeal carcinoma (NPC), and silencing FTO can effectively halt NPC growth and metastasis (Yang et al., 2024). Conversely, SPI1-induced downregulation of FTO in glioblastoma (GBM) promotes tumor growth by altering the processing of pri-miR-10a in an m6A-dependent manner (Zhang S. et al., 2022).

m6A reading proteins also exhibit contradictory effects in different tumors. In breast cancer (BC), overexpression of YTHDC1 is associated with a poor prognosis. YTHDC1 promotes nuclear export and enhances SMAD3 expression, which in turn activates the TGF-β signaling cascade, contributing to the metastasis of triple-negative breast cancer (TNBC) (Tan B. et al., 2022). On the other hand, a study by Yan et al. found that YTHDC1 is downregulated in bladder cancer tissues, where its reduced expression correlates with poor prognosis. The YTHDC1/GLUT3/RNF183 axis establishes a positive feedback loop that regulates bladder cancer progression and glucose metabolism (Yan et al., 2023).

Furthermore, enzymes related to m6A that typically play similar roles within the same tumor can exhibit conflicting effects. For instance, METTL14, which is underexpressed in colorectal cancer (CRC) and linked to a poor prognosis, reduces m6A levels on XIST and enhances YTHDF2-mediated XIST expression, thereby inhibiting tumor cell growth (Yang et al., 2020). Conversely, another study found that METTL3 is upregulated in CRC tissues compared to normal tissues. METTL3 overexpression accelerates tumor growth by activating the m6A-Hippo axis on CRB3 (Pan et al., 2022).

### 4.4 M6A enzymes with the same or similar role also exhibit contradictory roles in the same tumor

m6A enzymes with similar functions can exhibit contradictory roles within the same tumor. Clinical research has shown a negative correlation between METTL14-m6A levels and dysfunctional T cells in colorectal cancer (CRC) patients. The loss of RNA N6-adenosine methyltransferase METTL14 in tumor-associated macrophages (TAMs) can stimulate tumor formation and impair CD8^+^ T cell function (Dong et al., 2021). In a separate CRC study, METTL3 enhances GLUT1 translation in an m6A-dependent manner. Elevated METTL3 expression correlates with poor survival rates in CRC patients, suggesting it could be a valuable target for therapeutic intervention (Chen H. et al., 2021).

Similarly, m6A demethylases with comparable functions show contradictory roles in the same tumor. In pancreatic cancer, suppression of ALKBH5 significantly promotes the migration, invasion, and proliferation of pancreatic ductal adenocarcinoma (PDAC) cells both *in vitro* and *in vivo*, whereas ALKBH5 overexpression yields the opposite effects. Low ALKBH5 expression is associated with poor clinical outcomes in PDAC patients (Tang et al., 2020). In contrast, elevated expression of FTO in PDAC is linked to a worse prognosis, and silencing FTO halts tumor cell growth. FTO stabilizes PDGFC mRNA in an m6A-dependent manner, promoting cell proliferation (Tan Z. et al., 2022). FTO has also been implicated in gemcitabine resistance in pancreatic cancer (Lin K. et al., 2023). Additionally, m6A modification-induced upregulation of FZR1 translation has been shown to enhance gemcitabine resistance in PDAC (Su et al., 2023).

YTHDC1 knockdown has been found to increase lung cancer growth by elevating FSP1 protein levels, which control ferroptosis and facilitate tumor propagation (Yuan et al., 2023). M6A methyltransferase proteins also play crucial roles in tumor metastasis. For instance, IGF2BP3 is overexpressed in metastatic lung adenocarcinoma, and its elevated expression is associated with poor prognosis. IGF2BP3 activates Notch signaling in an m6A-dependent manner, triggering partial epithelial-mesenchymal transition (EMT) in lung adenocarcinoma cells (Yang X. et al., 2023).

Similarly, even within the same tumor, the same m6A-related enzyme can exhibit contradictory effects. For example, in bladder cancer, METTL14 is upregulated, leading to the promotion of lncDBET expression through METTL14-mediated m6A modification. Upregulated lncDBET activates the PPAR signaling pathway by directly interacting with FABP5, thereby promoting lipid metabolism in cancer cells and facilitating the malignant progression of bladder cancer (Liu P. et al., 2022). In contrast, another study on bladder cancer shows that METTL14 is downregulated in bladder tumor-initiating cells (TICs), and its deletion increases TIC self-renewal and bladder tumorigenesis (Gu et al., 2019).

A similar paradox is observed with the same demethylase in pancreatic cancer. ALKBH5 can maintain the stemness of pancreatic cancer cells and inhibit their sensitivity to gemcitabine (Zhang Y. et al., 2022). However, ALKBH5 can also induce m6A demethylation of KCNK15-AS1, thereby upregulating KCNK15-AS1, which binds to the 5′UTR of KCNK15 to suppress its translation, thus inhibiting the growth of pancreatic cancer cells (He et al., 2021).

Moreover, even the same methylation reader protein can exhibit conflicting effects within the same tumor. In an HBV-related hepatocellular carcinoma (HCC) model, YTHDF2 stabilizes MCM2 and MCM5 transcripts in an m6A-dependent manner (Yang Y. et al., 2023). Conversely, YTHDF2 overexpression inhibits HCC cell growth and proliferation by binding directly to the m6A modification site on the 3′-UTR of EGFR mRNA, promoting its degradation and halting tumor progression (Zhong et al., 2019).

## 5 M6A modification regulates the potential relationship of ferroptosis in tumor progression

A growing body of research has revealed the significant role that ferroptosis plays in various malignancies since its discovery in 2012. As a recently identified form of programmed cell death, ferroptosis offers a potential therapeutic avenue to prevent the development and metastasis of cancer (Stockwell, 2022). Ferroptosis is a complex process that requires coordinated signals from several organelles, including lysosomes, peroxisomes, and the endoplasmic reticulum. It is closely linked to oxidative damage and antioxidant defense mechanisms, which are key components of the common ferroptosis pathways. Specifically, iron accumulation precedes lipid peroxidation, ultimately leading to rupture of the cytoplasmic membrane (Chen X. et al., 2021). Ferroptosis is a non-apoptotic, oxidative, iron-dependent form of cell death, characterized by unique gene markers associated with lipid peroxidation and iron accumulation. The process can be regulated at multiple levels, particularly in the realm of epigenetics. Recent studies have increasingly highlighted a potential connection between ferroptosis and m6A modification in regulating tumor growth (Table 1).

**TABLE 1:** The roles of RNA m6A-regulated ferroptosis in various tumors.

Types	M6A regulators	Ferroptosis-related genes	M6A involved in ferroptosis pathway	Inhibits/promotes ferroptosis	Tumor types	Molecular regulatory mechanism	References
Writer	METTL3	FSP1	Antioxidant system	Inhibit	Non-small cell lung cancer (NSCLC)	In NSCLC,miR-4443 inhibits cispla- tin-induced ferroptosis by negatively regulating the level of METTL3 in- duced FSP1 m6A methylation, there-by conferring cisplatin resistance in NSCLC cells	Song et al. (2021)
Writer	METTL3	SLC7A11	Antioxidant system	Inhibit	Lung adenocarcinoma (LUAD)	In LUAD, METTL3 mediated m6A modification can stabilize SLC7A11 mRNA and promote its translation, thus promoting LUAD cell proliferation and inhibiting cell ferroptosis	Xu et al. (2022a)
Writer	METTL3	SLC7A11	Antioxidant system	Inhibit	Hepatoblastoma (HB)	In hepatoblastoma, METTL3-IGF2BP1 mediated m6A modifcation promotes the inhibition ofCCR4-NOT complex-mediated adenylate deadnylation, which enhances SLC7A11 mRNA stability and expression and inhibits tumor ferroptosis	Liu et al. (2022d)
Writer	METTL14	SLC7A11	Antioxidant system	Inhibit	Hepatocellular carcinoma (HCC)	In hypoxic conditions, METTL14 inhibits ferroptosis in hepatocelluar carcinoma cells through m6A-YTHDF2-mediated degradation of SLC7A11 mRNA at the 5′UTR which in turn promotes tumor development	Fan et al. (2021)
Writer	METTL14	GPX4	Antioxidant system	Promote	Endometrial carcinoma	In endometrial carcinoma, METTL14 overexpression promotes methylation modification via an m6A-YTHDF2-dependent mechanism, reducing GPX4 mRNA stability, increasing lipid peroxidation levels, and acceler- ating ferroptosis	Wang et al. (2023a)
Writer	METTL16	GPX4	Antioxidant system	Inhibit	Breast cancer (BC)	METTL16 stabilizes GPX4 mRNA, inhibits ferroptosis, and promotes malignant progression of breast cancer	Ye et al. (2023)
Writer	METTL16	ATF4	Iron and lipid metabolism	Inhibit	Cholangiocarcinoma (CC)	METTL16 enhances the expression of ATF4 mRNA, inhibits ferroptosis, and is associated with a poor progno- sis in patients with CC.	Zhao et al. (2025)
Writer	WTAP	NRF2	Antioxidant system	Inhibit	Bladder cancer	In bladder cancer, WTAP/YTHDF1 promotes cell viability of bladder cancer and inhibits erastin-induced ferroptosis by promoting the levels of the antioxidant factor NRF2 mRNA.	Wang et al. (2023b)
Eraser	ALKBH5	NRF2	Antioxidant system	Promote	Thyroid cancer	In thyroid cancer, ALKBH5 inhibits thyroid cancer progression by induc- ing ferroptosis through m6A-TIAM1-Nrf2/HO-1 axis	Li et al. (2023c)
Eraser	ALKBH5	SLC7A11	Antioxidant system	Promote	Non-small cell lung cancer (NSCLC)	In NSCLC, ALKBH5 is identifed to function as the tumor suppressor via m6A-mediated SLC7A11 mRNA and ferroptosis induction	Huang et al. (2024)
Eraser	FTO	SLC7A11	Antioxidant system	Inhibit	Glioblastoma (GBM)	In GBM,FTO promotes m6A-de- pendent SLC7A11 mRNA stability, which inhibits SLC7A11 dependent ferroptosis and facilitates GBM progression	Tian et al. (2025)
Eraser	FTO	SLC7A11	Antioxidant system	Promote	Rapillary thyroid cancer (PTC)	In PTC, FTO regulates PTC cell ferroptosis by mediating the m6 methylation of SLC7A11,which in turn promotes the degradation ofSLC7A11 mRNA, thereby inducing ferroptosis and attenu- ating tumor migration and inva- sion	Ji et al. (2022)
Eraser	FTO	SLC7A11 and GPX4	Antioxidant system	Inhibit	Colorectal cancer (CRC)	Elevated FTO expression induced SLC7A11 and GPX4 expression through an m6A-YTHDF2-de-pendent mechanism to resist ferroptosis in CRC cells	Qiao et al. (2024)
Reader	YTHDF1	TFRC	Iron metabolism	Promote	Hypopharyngeal squamous cell carcinoma (HPSCC)	In HPSCC, YTHDF1 methyl- transferase domain interacts with the 3′UTR and 5′UTR of TRFC mRNA, which positively regulates translation of m6A-modified TFRC mRNA and promotes tumor ferroptosis	Ye et al. (2020)
Reader	YTHDF2	ACSL4	Lipid metabolism	Inhibit	Gastric cancer (GC)	In hypoxic conditions, CBSLR interacts with YTHDF2 to form a CBSLR/YTHDF2/CBS signaling axis,decreasing the stability of CBS mRNA, leading to protein polyubiquitination and degradation of ACSL4 and inhibiting ferroptosis in GC cells	Yang et al. (2022)
Reader	YTHDF2	FSP1	Antioxidant system	Inhibit	Hepatocellular carcinoma (HCC)	In HCC, HDLBP regulates the splicing of the ferroptosis associ- ated lncFAL in a YTHDF2-de-pendent manner, which affects HCC susceptibility to ferroptosis by directly binding to FSPl and competitively inhibiting its ubiq- uitination	Yuan et al. (2022)
Reader	YTHDC2	SLC3A2	Antioxidant system	Promote	Lung adenocarcinoma (LUAD)	In LUAD, overexpression of YTHDC2 promotes m6A-depen- dent SLC3A2 mRNA degrada-tion,which inhibits cystine trans- port and activates ferroptosis	Ma et al. (2021b)
Reader	IGF2BP3	GPX4	Antioxidant system	Inhibit	Glioma	IGF2BP3-mediated m6A modifica- tion can stabilize GPX4 mRNA andpromote its translation, thus inhibiting cell ferroptosis and promoting glioma cell growth and survival	Deng et al. (2024)
Reader	IGF2BP3	NRF2	Antioxidant system	Inhibit	Hepatocellular carcinoma (HCC)	In HCC, IGF2BP3 inhibits sorafenib induced ferroptosis by promoting NRF2 mRNA stability	Lu et al. (2022)

### 5.1 M6A methyltransferases and ferroptosis

The function and underlying mechanisms of METTL3 in ferroptosis have garnered increasing attention in recent years. Song et al. demonstrated that exosomes derived from cisplatin-resistant NSCLC tumors exhibited elevated levels of microRNA-4443 (miR-4443), and this upregulation was associated with chemoresistance to cisplatin in NSCLC. Further investigation revealed that METTL3 can mediate m6A modification of FSP1, reducing its expression, while miR-4443 interacts with the 3′UTR of METTL3, influencing intracellular superoxide, ROS, and ferroptosis, as well as FSP1 expression. This interaction ultimately inhibits ferroptosis. Interestingly, the m6A modification of FSP1 can be increased by reducing the expression of exosomal miR-4443, providing a novel strategy to overcome chemoresistance in NSCLC and offering potential therapeutic approaches targeting ferroptosis (Song et al., 2021).

Furthermore, the cystine/glutamate antiporter system Xc-is another important pathway in ferroptosis, and blocking it can induce ferroptosis. The system is primarily composed of SLC7A11 and solute carrier family 3 member 2 (SLC3A2). METTL3 plays a crucial role in regulating lung adenocarcinoma (LUAD) and hepatoblastoma. Xu et al. found that METTL3-mediated m6A modification enhances the stability of SLC7A11 mRNA, promoting its translation and suppressing ferroptosis, thereby facilitating LUAD cell proliferation (Xu Y. et al., 2022). Similarly, in hepatoblastoma, METTL3-mediated m6A modification increases the stability and expression of SLC7A11 mRNA, conferring resistance to ferroptosis and accelerating tumor growth (Liu L. et al., 2022).

In the m6A writer complex, METTL14, a key enzyme, forms an asymmetric dimer with METTL3. By binding to target RNA, the RNA-binding scaffold of METTL14 in this dimer enhances METTL3’s catalytic activity. Recent studies have further highlighted the critical role of METTL14 in the development and progression of ferroptosis. Both breast cancer (BC) and hepatocellular carcinoma (HCC) that express the human epidermal growth factor receptor 2 (HER2) are associated with METTL14. When METTL14 levels are reduced, fibroblast growth factor receptor 4 (FGFR4) expression increases. YTHDC2 recognizes and binds to the m6A site of FGFR4 mRNA, stabilizing it. This stabilization, coupled with the phosphorylation of glycogen synthase kinase 3β (GSK3β) and the activation of the β-catenin/TCF4 signaling pathway, upregulates FGFR4 expression. This mechanism prevents ferroptosis and enhances resistance to anti-HER2 therapy (Zou et al., 2022).

In hypoxia-mediated HCC, SLC7A11 has been identified as a direct target of METTL14. METTL14-induced m6A modification in the 5′UTR of SLC7A11 enhances its degradation through a YTHDF2-dependent mechanism. Under hypoxic conditions, however, METTL14 expression is repressed, preventing HCC cells from undergoing ferroptosis (Fan et al., 2021). Furthermore, METTL14-mediated regulation of ferroptosis plays a significant role in drug resistance in tumors. In endometrial cancer, PRMT3-mediated arginine methylation of METTL14 promotes tumor progression and treatment resistance. Specifically, inhibition of PRMT3 leads to METTL14 overexpression, which accelerates ferroptosis, decreases the stability of GPX4 mRNA, increases lipid peroxidation, and overcomes resistance to radiotherapy and platinum-based treatments via a m6A-YTHDF2-dependent mechanism (Wang Y. et al., 2023).

Recent studies have increasingly highlighted the role of Wilms Tumor 1-Associated Protein (WTAP) as a key player in human cancers, including hepatocellular carcinoma (HCC) and bladder cancer. Chen et al. demonstrated that ferroptosis is inhibited by WTAP-mediated m6A modification of circCMTM3, which contributes to HCC progression. Mechanistically, WTAP-mediated m6A modification of circCMTM3 enhances its interaction with IGF2BP1, leading to increased PARK7 expression and stability, which suppresses ferroptosis in HCC (Chen S. et al., 2023). Additionally, WTAP upregulates ATG5 post-transcriptionally in an m6A-YTHDC2-dependent manner, promoting ferroptosis in HCC and inhibiting tumor progression (Li et al., 2024). In bladder cancer, Wang et al. reported that ferroptosis is inhibited and WTAP expression is elevated. Through a YTHDF1-m6A-dependent mechanism, WTAP induces m6A modification at the 3′UTR of erythroid-derived 2-like 2 (NFE2L2/NRF2) mRNA, thereby enhancing NRF2 mRNA stability (Wang K. et al., 2023).

Current research on methyltransferases, particularly METTL3, METTL14, and WTAP, primarily focuses on their roles in regulating ferroptosis and tumor growth. Through direct or indirect regulation of ferroptosis-related proteins, these methyltransferases influence tumor progression. However, further investigation is needed to fully elucidate the precise mechanisms by which different methyltransferases modulate ferroptosis in various malignancies. Recent studies suggest that METTL16 may play a crucial role in regulating macrophage antioxidant levels. METTL16 binds to the triple-helix structure at the 3′end of metastasis-associated lung adenocarcinoma transcript 1 (MALAT1), forming a complex that facilitates m6A modification of the 3′UTR of methionine adenosyltransferase 2A (MAT2A) mRNA. This modification leads to the degradation of MALAT1, which in turn decreases MAT2A methylation mediated by METTL16, increases the ratio of glutathione to glutathione disulfide (GSH/GSSG), and reduces reactive oxygen species (ROS) levels. While the role of METTL16 in ferroptosis during tumor progression is not yet fully understood, further studies are required to determine whether METTL16 influences tumor growth by modulating cellular antioxidant capacity. Notably, previous studies have identified m6A-modified genes (such as METTL16, METTL5, METTL3, FMR1, and HNRNPC) and ferroptosis-related long non-coding RNAs (lncRNAs), which can be utilized in bioinformatics approaches to predict and evaluate risk models for ovarian cancer (Zheng et al., 2021). Although some research has examined the relationship between ferroptosis-related lncRNAs and the immunotherapeutic efficacy of m6A-related genes (Xie et al., 2022; Huang et al., 2022), few studies have focused on specific targets or pathways involving m6A methyltransferases and ferroptosis in immunotherapy. Further research across various cancer types is essential to enhance the efficacy of tumor immunotherapy.

The main targets of m6A methyltransferase-mediated ferroptosis are GPX4 and SLC7A11. However, the influence of m6A methylation on tumor growth via ferroptosis remains a topic of considerable debate. m6A methyltransferase-mediated ferroptosis can either promote or inhibit tumor growth, depending on the context, highlighting its complex and multifaceted role in cancer. For example, in non-small cell lung cancer (NSCLC), METTL3 acts as a catalyst for ferroptosis by targeting GPX4 and SLC7A11. However, METTL3 can also inhibit ferroptosis by downregulating SLC7A11 in lung cancer and glioblastoma (GBM). Additionally, METTL14 demonstrates a dual role in ferroptosis. In endometrial cancer, METTL14 overexpression promotes ferroptosis by destabilizing GPX4 mRNA (Wang Y. et al., 2023). Conversely, in HER2-positive breast cancer (BC), METTL14 inhibits ferroptosis. The specific pathways linking METTL14 to ferroptosis, however, remain unclear.

**FIGURE 3:**
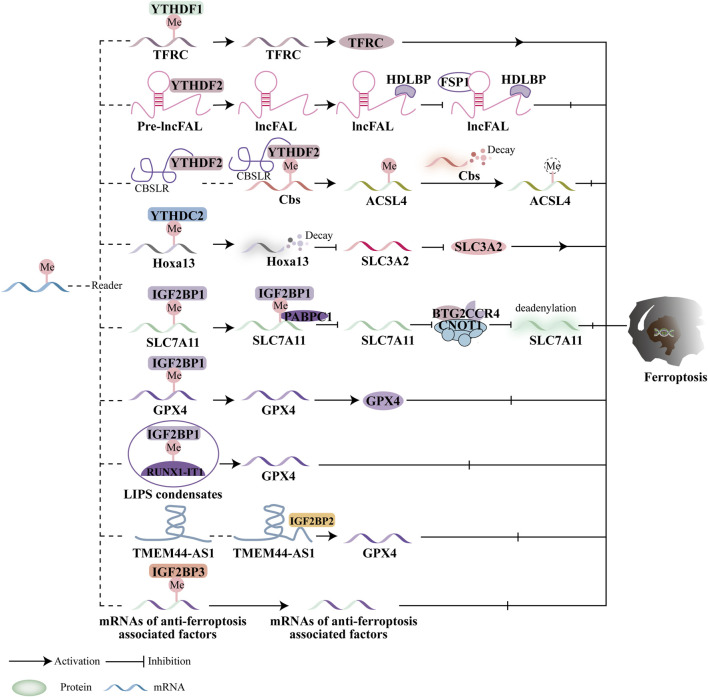
Ferroptosis and the function of m6A methyltransferases. By modulating downstream targets in a m6A-dependent way, the writers control ferroptosis. Ferroptosis is regulated by downstream targets mediated by METTL3, WTAP, and METTL14.

Overall, the roles of METTL3 and METTL14 in regulating GPX4 and SLC7A11 are central to current research on m6A methyltransferase-mediated ferroptosis and its impact on tumor growth. While WTAP has been shown to inhibit ferroptosis in bladder cancer and HCC, the precise relationship between WTAP and ferroptosis remains poorly understood (Figure 3).

### 5.2 M6A demethylase and ferroptosis

**FIGURE 4:**
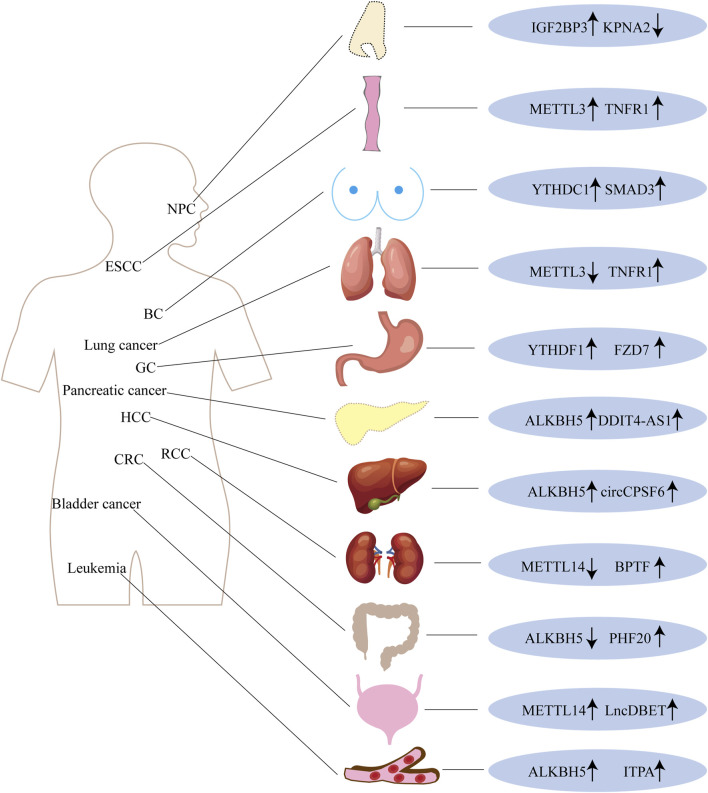
Ferroptosis and the function of m6A demethylases. In a m6A-dependent manner, the erasers modulate ferroptosis by mediating downstream targets. By modulating downstream targets, ferroptosis can be regulated by FTO and ALKBH5.

As illustrated in Figure 4, the activity of m6A demethylases is closely linked to ferroptosis, with an expanding body of research highlighting the significant role of m6A demethylation-mediated ferroptosis in tumor progression. Specifically, ALKBH5 is a critical regulator in the development of several cancers, including thyroid cancer, hypopharyngeal carcinoma, non-small cell lung cancer (NSCLC), and glioblastoma (GBM). According to Wei et al., ALKBH5 inhibits the progression of thyroid cancer by inducing ferroptosis through the TIAM1-NRF2/HO-1 axis (Li W. et al., 2023). In hypopharyngeal squamous cell carcinoma (HPSCC), ALKBH5-mediated m6A demethylation regulates the post-transcriptional modulation of NFE2L2/NRF2. IGF2BP2, the m6A reader, plays a crucial role in stabilizing m6A-modified NFE2L2/NRF2. Knocking down ALKBH5 increases NFE2L2/NRF2 expression by promoting IGF2BP2 binding to the 3′UTR of NFE2L2/NRF2 mRNA, which leads to elevated GPX4 expression and the inhibition of ferroptosis (Ye et al., 2022). Furthermore, upregulation of ALKBH5 through m6A demethylation of SLC7A11 promotes ferroptosis in NSCLC cells, ultimately suppressing NSCLC progression (Huang et al., 2024). Additionally, EGFR has been shown to promote the nuclear localization of m6A demethylase ALKBH5, which reduces m6A levels and protects GBM cells from ferroptosis (Lv et al., 2023).

Furthermore, FTO may directly modify gene targets related to ferroptosis, thereby influencing tumor development. Studies by Wang et al. demonstrate that FTO decreases the stability of ACSL3 and GPX4 mRNA in oral squamous cell carcinoma (OSCC) by demethylating m6A modifications, which reduces the expression of ACSL3 and GPX4, promotes ferroptosis, and impedes OSCC progression (Wang Z. et al., 2023). Additionally, research suggests that FTO downregulates SLC7A11 in an m6A-dependent manner to inhibit thyroid cancer development through ferroptosis (Ji et al., 2022). In colorectal cancer (CRC), FTO overexpression inhibits ferroptosis by upregulating SLC7A11 and GPX4, thereby promoting CRC carcinogenesis through an m6A-YTHDF2-dependent mechanism (Qiao et al., 2024).

Despite these findings, there is still limited information on the therapeutic effectiveness of m6A demethylase-mediated ferroptosis in malignancies. Moreover, the impact of m6A demethylase-mediated ferroptosis on tumor growth remains controversial. The functions of m6A demethylases are paradoxical, as FTO and ALKBH5 can either accelerate or prevent ferroptosis. For instance, ALKBH5 or FTO inhibits ferroptosis in hypopharyngeal carcinoma or CRC by targeting GPX4 or SLC7A11. However, by focusing on genes linked to lipid peroxidation, both ALKBH5 and FTO can also induce ferroptosis in non-small cell lung cancer (NSCLC) and oral squamous cell carcinoma. The status and role of m6A mRNA can vary depending on the specific disease and its stage. Further research into the signaling pathways of ferroptosis and m6A demethylases is crucial to better understanding their therapeutic potential.

### 5.3 M6A reading protein and ferroptosis

**FIGURE 5:**
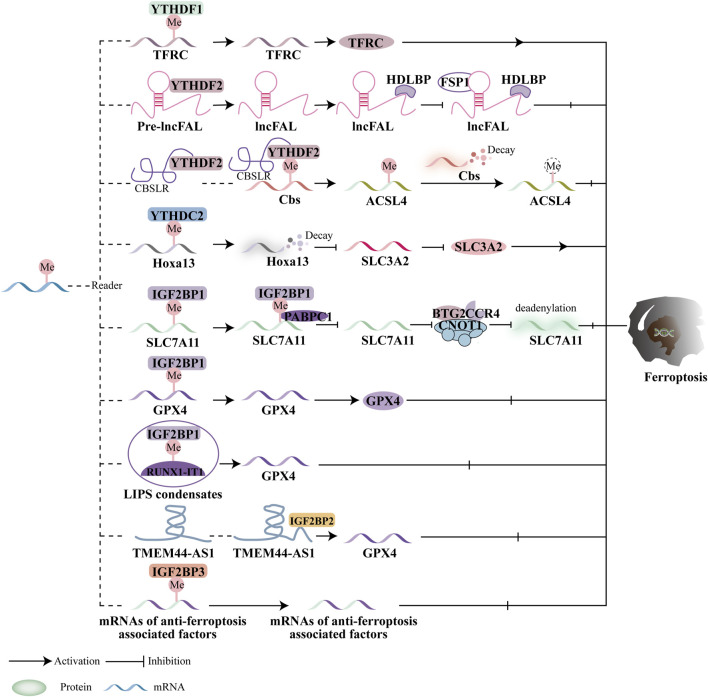
Ferroptosis and the function of m6A reader proteins. In a m6A-dependent way, readers mediate downstream targets to control ferroptosis. The YTH family and IGF2BP are significant in either stimulating or suppressing ferroptosis based on mediating different target proteins.

m6A reader proteins can mediate the regulation of ferroptosis in downstream targets (Figure 5). YTHDF1 is closely associated with iron metabolism in hypopharyngeal squamous cell carcinoma (HPSCC) patients. The 3′and 5′UTRs of transferrin receptor (TFRC) mRNA interact with the methyltransferase domain of YTHDF1, which promotes the translation of TFRC mRNA, increasing intracellular iron concentration, Fe^2+^ levels, and reactive oxygen species (ROS) (Ye et al., 2020). Recently, the involvement of lncRNAs with m6A alterations in cancer progression has garnered significant attention. Yang et al. showed that HIF-1α induces the production of lncRNA-CBSLR, which recruits YTHDF2 to form the CBSLR/YTHDF2/CBS complex. This complex enhances YTHDF2’s binding to m6A sites in the CBS coding region, thereby reducing CBS mRNA stability. The downregulation of CBS expression inhibits ferroptosis in gastric cancer (GC) cells by promoting the degradation of ACSL4 (Yang et al., 2022). A recent study also highlighted the important role of YTHDF2 in hepatocellular carcinoma (HCC). Specifically, this work focuses on how high-density lipoprotein binding protein (HDLBP) modulates an HCC patient’s sensitivity to ferroptosis. The plexin B2 gene encodes lncFAL, a long non-coding RNA that can bind to HDLBP. Additionally, lncFAL interacts with YTHDF2 to promote its splicing, and by directly binding to FSP1, lncFAL reduces ferroptosis susceptibility (Yuan et al., 2022). Moreover, Ma et al. discovered that SLC3A2 is critical for YTHDC2-regulated ferroptosis. SLC7A11 and SLC3A2 are key components of cysteine/glutamate reverse transporters. YTHDC2 recognizes the m6A modification in the 3′-UTR of HOXA13 mRNA, destabilizing the mRNA and suppressing the production of SLC3A2. This reduces LUAD oxidation and inhibits tumor growth (Ma et al., 2021b). It is noteworthy that some lncRNAs related to ferroptosis have been reported to interact with m6A-related genes (Xie et al., 2022; Huang et al., 2022). However, the relationship between ferroptosis-related lncRNAs and m6A modification in predicting immunotherapeutic outcomes remains poorly understood. Further investigation is necessary to explore how m6A-modified ferroptosis influences tumor immune evasion, especially given the potential roles of both ferroptosis and m6A in malignancy progression.

Moreover, IGF2BP1 regulates ferroptosis by directly recognizing and binding to the m6A modification sites of key ferroptosis-related proteins. In human hepatocellular carcinoma (HCC) and hepatoblastoma cells, IGF2BP1 binds to the 3′UTR of SLC7A11 mRNA, enhancing its stability and preventing deadenylation. Additionally, IGF2BP1 competes with cytoplasmic poly(A)-binding protein-1 (PABPC1) to inhibit the formation of the BTG2/CCR4-NOT complex, which otherwise promotes deadenylation of SLC7A11 mRNA and accelerates ferroptosis (Liu L. et al., 2022). RUNX1-Intron Transcript 1 (RUNX1-IT1), which directly binds to IGF2BP1 and promotes liquid-liquid phase separation (LLPS), is more abundant in human bladder cancer (BC) tissues. This results in increased occupancy of IGF2BP1 on GPX4 mRNA, stabilizing it, inhibiting ferroptosis, and supporting BC progression (Wang S. et al., 2023). While the precise role of IGF2BP2 in ferroptosis remains unclear, it is known that lncRNAs play a significant role in cancer progression. The lncRNA TMEM44-AS1, for instance, can inhibit ferroptosis and stabilize GPX4 mRNA, thus promoting the malignant growth of esophageal squamous cell carcinoma (ESCC) by binding to the RNA-binding protein IGF2BP2 (Yang R. et al., 2023). IGF2BP3, which is highly expressed in lung adenocarcinoma (LUAD), inhibits ferroptosis by binding to key ferroptosis-related proteins such as GPX4, SLC3A2, ACSL3, and FTH1, making it a promising target for anticancer therapies (Xu X. et al., 2022). Studies have also indicated that IGF2BP3 serves as a poor prognostic marker in gliomas. Specifically, IGF2BP3 binds to motifs on GPX4 mRNA to upregulate GPX4 protein expression, thereby inhibiting ferroptosis (Deng et al., 2024). Additionally, IGF2BP3 is involved in the initiation of a circRNF10/ZBTB48/IGF2BP3 positive feedback loop in gliomas, leading to reduced Fe^2+^ accumulation and activation of ferroptosis defense mechanisms, which promotes glioblastoma (GBM) progression (Wang C. et al., 2023). Furthermore, Wu et al. evaluated the prognosis and progression of pancreatic cancer patients using a predictive model that incorporated ferroptosis regulators and m6A methyltransferases, including IGF2BP2, IGF2BP3, and METTL16 (Wu et al., 2022).

Current research primarily focuses on the YTH domain family and IGF2BPs in relation to ferroptosis and cancer treatment efficacy. For instance, the m6A reader proteins of the YTH domain family, such as YTHDF1 and YTHDC2, regulate ferroptosis by targeting key molecules like System Xc- (SLC3A2), the Fe^2+^ pool, and the subunits of ACSL4. Additionally, anti-ferroptotic factors such as SLC7A11 and GPX4 are regulated by IGF2BP1 and IGF2BP3. However, there is limited research on the roles of YTHDF3 and IGF2BP2 in ferroptosis and cancer, and further investigation is necessary to determine whether these proteins contribute to ferroptosis regulation. The impact of m6A reader proteins on ferroptosis and tumor progression remains controversial, with these proteins displaying contradictory effects. For example, YTHDF1 and YTHDC2 act as promoters of ferroptosis in hypopharyngeal squamous cell carcinoma (HPSCC) and lung adenocarcinoma (LUAD). In contrast, other m6A reader proteins appear to suppress ferroptosis in gastric cancer (GC), hepatocellular carcinoma (HCC), esophageal squamous cell carcinoma (ESCC), glioblastoma (GBM), and bladder cancer (BC).

In conclusion, most current research on the effects of YTH domain proteins and IGF2BPs on lipid peroxidation or antioxidant enzymes primarily focuses on m6A reader protein-mediated ferroptosis. However, the roles of nuclear m6A-binding proteins and their potential involvement in regulating tumor progression through ferroptosis remain largely unexplored.

## 6 Potential therapeutic applications of m6A-modified ferroptosis in cancer

M6A modification, a common type of RNA modification, plays a crucial role in numerous biological processes. Ferroptosis, an iron-dependent form of cell death, is emerging as a promising therapeutic strategy to inhibit cancer development and metastasis. Recent studies have highlighted that m6A alterations significantly influence key biological characteristics of cancer, including drug resistance, metastasis, and cell proliferation. The growing understanding of ferroptosis and m6A-based anti-tumor approaches suggests their potential in addressing challenges in cancer therapy. Therefore, exploring the relationship between m6A modifications and ferroptosis in cancer, along with their therapeutic implications, is essential (Table 2).

**TABLE 2:** The potential clinical application of m6A-modified ferroptosis.

Types	M6A regulators	Target gene	Clinical application	Tumor types	Molecular regulatory mechanism	References
Writer	METLL3 WTAP	ACSL5	Lenvatinib resistance	Hepatocellular carcinoma (HCC)	Under normoxic and hypoxic conditions, METTL3 and WTAP regulates ferro- ptosis through the PPARGC1A/BAM-BI/ACSL5 axis, promoting HCC progression and lenvatinib resistance	Zhang et al. (2023)
Writer	METTL3	SLC7A11	Cisplatin resistance	Gastric cancer (GC)	In GC,METTL3-induced m6A modification and YTHDC1-induced stability of FAM120AmRNA enhance the expression ofFAM120A, which inhibits ferroptosis by binding to SLC7A11 mRNA and enhancing its stability, while the deficiency of FAM120A increases cisplatin sensitivity by promoting ferroptosis	Niu et al. (2024)
Writer	METLL3	miR-4443	Cisplatin resistance	Non-small cell lung cancer (NSCLC)	In NSCLC, miR-4443 regulates the expres- sion ofFSP1 through METTL3 in an m6A-dependent manner, thereby inhibiting FSP1-mediated ferroptosis induced by cisplatin treatment *in vitro* and promoting tumor growth *in vivo*	Song et al. (2021)
Writer	METLL3	SLC7A11	Radioresistance	Nasopharyngeal carcinoma (NPC)	In NPC, METTL3 stabilizes SLC7A11 mRNA, thereby inhibiting radiation-induced ferroptosis and ultimately inducing radiore-sistance in NPC	Dai et al. (2025)
Writer	METLL3 IGF2BP2	SLC7A11	Radioresistance	Hepatocellular carcinoma (HCC)	In HCC, METTL3 regulates the stability of SLC7A11 mRNA in an m6A/IGF2BP2-de- pendent manner, which can modulate the sensitivity of hepatocellular carcinoma to radiotherapy	Zhang et al. (2025)
Writer	METTL14	FGFR4	Anti-HER2 therapy	Breast cancer (BC)	In BC,METTL14-mediated FGFR4 reduces resistance to anti-HER2 therapy by activat- ing ferroptosis through the inhibition of glutathione synthesis and the blockade of Fe^2+^ efflux efficiency	Zou et al. (2022)
Eraser	M6A erasers	PD-L1	Immunotherapy	Acute myeloid leukemia (AML)	In leukemia GNRa-CSP12 abolishes endogenous Fe^2+^-dependent m6A demeth- ylase activity, resulting in global m6A hypomethylation and post-transcriptional regulation of downstream genes involved in glycolysis, hypoxia, and immune check- point pathways	Du et al. (2021)
Eraser	FTO	OTUB1	Radioresistance	Nasopharyngeal carcinoma (NPC)	FTO functions as an m6A demethylase to erase the m6A modification of the OTUB1 transcript and promote the expression of OTUB1, thereby inhibiting radiation-in duced ferroptosis in cells and finally triggering the radiotherapy resistance of NPC	Huang et al. (2023)
Reader	IGF2BP3	NRF2	Sorafenib resistance	Hepatocellular carcinoma (HCC)	IGF2BP3, as an m6Areader, promotes the stability of NRF2 mRNA by recognizing its m6A modification, thereby increasing sorafenib resistance in HCC.	Lu et al. (2022)
Reader	YTHDF1	PD-L1	Immunotherapy	Prostate cancer (PCa)	In PCa,YTHDF1 promotes functional PD-L1 partially by enhancing its transcrip- tional stability, which is essential for prostate cancer (PCa) cells to evade effector T cell cytotoxicity and ferroptosis mediated by CD8^+^ T cells	Wang et al. (2024a)
Reader	YTHDF2	CBS	Chemoresistance	Gastric cancer (GC)	CBSLR interacts with YTHDF2 to form a CBSLR/YTHDF2/CBS signaling axis that decreases the stability of CBS mRNA, leading to the degradation of ACSL4, which contributes to ferroptosis resistance in GC cells	Yang et al. (2022)
Reader	YTHDF2	SLC2A3	Immunotherapy	Lung adenocarcinoma (LUAD)	NETs inhibit ferroptosis and CD8 (+) T cell activity through YTHDF2-mediated degradation of SLC2A3 mRNA, demonstrating a mechanism by which NETs alter the tumor microenvironment to support LUAD growth	Xu et al. (2025)

Radiation therapy is a widely used treatment for cancer; however, its effectiveness is often compromised by tumor cells’ ability to tolerate it. Several studies have shown that radiation therapy can induce ferroptosis in cancer cells, while the inactivation of ferroptosis contributes to radiation resistance (Mao et al., 2024; Koppula et al., 2022; Chen Q. et al., 2023). The primary biological effects associated with tumor elimination through radiation include oxidative stress and ferroptosis. Inducing ferroptosis presents a potential strategy to overcome radiation resistance. For example, radiation therapy has been shown to induce ferroptosis in various cancer types, including non-small cell lung cancer (NSCLC), ovarian cancer, fibrosarcoma, adenocarcinoma, and glioma (Zhang et al., 2024; Liu et al., 2023). Moreover, while ferroptosis inhibitors counteract radiation therapy, ferroptosis inducers—such as the GPX4 inhibitor RSL3 and the system Xc-inhibitor erastin—enhance the therapeutic effects of radiation. In prostate cancer (PCa) patients undergoing radical radiation therapy, predictive indices of ferroptosis-related genes have been linked to biochemical recurrence and radiation resistance (Feng et al., 2022). Further evidence suggests that radiation therapy can promote ferroptosis in cancer patients, with heightened ferroptosis correlating with improved survival and increased sensitivity to irradiation (Lei et al., 2020). Key molecules like SLC7A11 and GPX4 are crucial in radiotherapy-induced ferroptosis, as m6A modification regulates several ferroptosis-related RNAs, including GPX4 and SLC7A11. These molecules influence iron metabolism and reactive oxygen species (ROS) production, thus modulating the response of tumor cells to radiation therapy. For instance, as an m6A demethylase, FTO inhibits radiation-induced ferroptosis, thereby enhancing the radioresistance of nasopharyngeal carcinoma (NPC) cells (Huang et al., 2023). Tumor cells’ radiosensitivity is closely linked to their susceptibility to ferroptosis, a process modulated by dynamic m6A modifications regulated by m6A regulators.

Immunotherapy has emerged as a key cancer treatment strategy by activating the body’s immune system to target and eliminate tumor cells. Recent studies indicate that m6A modification plays a crucial role in regulating the effectiveness of immunotherapy (Boulias and Greer, 2023; Wang L. et al., 2023). As an important epigenetic regulator, m6A modification influences the growth and metabolism of tumor cells, which in turn regulates ferroptosis, a novel form of cell death. By modulating m6A modifications, the iron metabolism and reactive oxygen species (ROS) production in tumor cells are altered, thereby influencing the mechanisms of cell death and making them more susceptible to therapeutic intervention. In the context of cancer immunotherapy, m6A modification can also affect the immune escape mechanisms of tumor cells, influencing their immunogenicity and responsiveness to immunotherapy. For example, through the m6A-PD-L1 axis, the m6A reader YTHDF1 has been shown to reduce ferroptosis and impair CD8^+^ T cell-mediated anti-tumor immunity in prostate cancer (PCa) (Wang Y. et al., 2024). Furthermore, the expression profiles of m6A regulators can predict outcomes in small cell lung cancer, including the effectiveness of adjuvant chemotherapy and anti-PD-1 immunotherapy (Zhang et al., 2021b; Zhang et al., 2021c). In acute myeloid leukemia (AML), the activity of endogenous Fe^2+^-dependent m6A demethylases is suppressed by GNRa-CSP12, leading to widespread m6A hypomethylation and post-transcriptional regulation of genes involved in immune checkpoint pathways, glycolysis, and hypoxia. The combination of GNRa-CSP12 and tyrosine kinase inhibitors (TKIs) effectively removes the m6A-mediated TKI resistance phenotype, suggesting that GNRa-CSP12 may serve as a valuable therapeutic agent to enhance the efficacy of leukemia immunotherapy (Du et al., 2021). These findings underscore the potential role of m6A modification in modulating the effectiveness of tumor immunotherapy.

Drug resistance in cancer cells is closely linked to m6A alteration. One of the major challenges in cancer treatment is overcoming drug resistance, and recent studies suggest that m6A modification plays a critical role in determining the sensitivity of tumor cells to chemotherapeutic drugs (Deng et al., 2023; Li F. et al., 2023). For example, nasopharyngeal carcinoma (NPC) exhibits elevated levels of METTL5/TRMT112 and its m6A modification at 18S rRNA position 1832 (m6A1832), which enhances neoplastic transformation both *in vitro* and *in vivo*. However, when the catalytic activity of METTL5 is lost, it paradoxically promotes NPC tumorigenesis and chemoresistance (Chen B. et al., 2023). Additionally, TEAD4 serves as an independent prognostic indicator of poor outcome and cisplatin resistance in NPC. YTHDF2 recognizes WTAP-mediated m6A methylation of TEAD4, promoting its stability and aberrant upregulation, which in turn facilitates NPC migration, invasion, and cisplatin resistance (Wang YQ. et al., 2023). In esophageal squamous cell carcinoma (ESCC), the m6A demethylase FTO stabilizes the long non-coding RNA LINK-A, promoting cell proliferation and chemoresistance (Nan et al., 2023). Moreover, many anticancer drugs exert their therapeutic effects by inducing ferroptosis in tumor cells, and both ferroptosis and m6A molecular regulators play key roles in mitigating chemoresistance. The combination of m6A modification and ferroptosis offers a promising approach to enhance the efficacy of anticancer drugs. m6A regulation directly impacts the sensitivity of tumor cells to drug-induced ferroptosis, improving the overall response to chemotherapy. For instance, Song et al. demonstrated that exosomal miR-4443, through METTL3-mediated regulation of FSP1 m6A modification, enhances cisplatin resistance in non-small cell lung cancer (NSCLC) via the m6A-ferroptosis axis (Song et al., 2021).

Numerous studies have demonstrated that m6A alteration modulates tumor cell sensitivity to anticancer drug-induced ferroptosis by regulating key genes associated with ferroptosis, which in turn influences chemotherapy efficacy and drug resistance mechanisms. For example, m6A hypomethylation regulates FGFR4 phosphorylation of GSK-3β, activating the β-catenin/TCF4 signaling pathway to confer resistance to anti-HER2 therapy. Inhibition of FGFR4 enhances the sensitivity of drug-resistant breast cancer (BC) cells to anti-HER2 treatment (Zou et al., 2022). In hepatocellular carcinoma (HCC), IGF2BP3 prevents ferroptosis following sorafenib treatment by modifying m6A and increasing the stability of NRF2 mRNA, which is closely linked to tumor growth and poor prognosis in HCC (Lu et al., 2022). In cisplatin-resistant gastric cancer (GC) tissues, upregulation of FAM120A expression was positively correlated with poor prognosis in GC patients. FAM120A bound to SLC7A11 mRNA, enhancing its stability and promoting cisplatin resistance. This regulation was induced by METTL3 and YTHDC1-mediated m6A modifications (Niu et al., 2024).

A recent study on hepatocellular carcinoma (HCC) found that ferroptosis is regulated by the PPARGC1A/BAMBI/ACSL5 axis, with METTL3 and WTAP inhibiting PPARGC1A in an m6A- and YTHDF2-dependent manner. This regulation promotes lenvatinib resistance and HCC progression (Zhang et al., 2023). The relationship between ferroptosis and m6A modification offers potential for identifying novel therapeutic targets and provides a fresh theoretical framework for clinical application. For example, hypoxia-induced lncRNA-CBSLR can regulate ferroptosis and chemoresistance in gastric cancer (GC) cells by reducing ferroptosis levels, thus promoting chemoresistance. This process is also dependent on m6A and YTHDF2. GC patients with high CBSLR and low CBS levels exhibit poorer chemotherapy responsiveness and worse clinical outcomes. Therefore, selecting appropriate chemotherapeutic agents based on CBSLR/CBS expression levels may optimize treatment efficacy (Yang et al., 2022).

In conclusion, m6A-mediated ferroptosis plays a pivotal role in cancer management. By regulating ferroptosis through m6A alterations, tumor cells can increase their sensitivity to radiation and immunotherapy, reduce drug resistance, and provide new therapeutic targets and strategies for cancer treatment. Although recent research has made significant strides, further studies are needed to fully elucidate the specific mechanisms of m6A-driven ferroptosis in cancer therapy and to better understand the interplay between m6A modifications and ferroptosis. Beyond m6A, other irreversible chemical modifications in DNA, RNA, and proteins—such as DNA methylation, m1A, m5C, ubiquitination, phosphorylation, acetylation, and glycosylation—may also influence ferroptosis. However, the extent to which these epigenetic changes affect malignancies remains to be determined.

## 7 Conclusion

m6A modification is regulated by a dynamic interplay between m6A methyltransferases, demethylases, and reader proteins, with the potential involvement of other m6A modifiers. Recent studies suggest that m6A influences ferroptosis through both mRNA and non-coding RNA, impacting the development and progression of cancer. However, the role of m6A in ferroptosis remains controversial, reflecting its complex involvement in various cancers. For instance, METTL3 acts as a promoter of ferroptosis in esophageal squamous cell carcinoma (ESCC) (Wang et al., 2021), while WTAP suppresses ferroptosis in hepatocellular carcinoma (HCC) (Li et al., 2024). Similarly, the m6A demethylase FTO promotes ferroptosis in nasopharyngeal carcinoma (NPC) (Yang et al., 2024), but inhibits it in glioblastoma (GBM) (Zhang S. et al., 2022). These contrasting roles may be explained by the different physiological contexts, as the function of m6A is influenced by both its microenvironment and upstream/downstream regulatory networks. Furthermore, m6A-related proteins are not limited to methylation regulation—they may exert opposing effects depending on the specific reader and writer proteins involved and the target genes they regulate. The effects of m6A alteration are context-dependent, modulating ferroptosis in both stimulating and inhibiting directions, depending on factors such as the relative activity of methyltransferases and demethylases, the specific m6A readers, the type of RNA (mRNA or ncRNA), and the characteristics of the disease. Additionally, while m6A methyltransferases like METTL16 have received limited attention, they are emerging as critical regulators of ferroptosis in cancer. For example, METTL16 has been shown to inhibit ferroptosis in HCC cells, promoting tumor progression, and its high expression correlates with poor prognosis in HCC patients (Wang J. et al., 2024).

Recent studies have demonstrated a close relationship between m6A regulatory mechanisms and ferroptosis. m6A methylation is capable of influencing the expression of genes related to iron metabolism, thereby modulating cellular sensitivity to ferroptosis (Zhao et al., 2025; Tian et al., 2025; Ye et al., 2023).This discovery provides a new perspective for anticancer therapies, particularly in the treatment of drug-resistant tumors, as m6A represents a potentially controllable target with significant therapeutic implications (Dai et al., 2025; Sun et al., 2025; Xu et al., 2025; Zhang et al., 2025). We propose that future research should focus on elucidating the specific molecular mechanisms linking m6A and ferroptosis, and exploring its clinical feasibility as a novel cancer treatment strategy. This includes the development of specific drugs or small molecules to regulate m6A levels, thereby enhancing the antitumor effects induced by ferroptosis.Overall, understanding the intricate relationship between ferroptosis and m6A modification is crucial for deciphering tumorigenesis and could pave the way for novel therapeutic strategies. m6A-related targets hold potential as key players in cancer treatment, prognosis, and overcoming therapeutic resistance.

## Glossary

m6A: N6-methyladenosine methylation
MTC: methyltransferase complex
RBM15/15B: RNA-binding motif protein 15/15B
WTAP: Wilms Tumor 1-Associating Protein
VIRMA: vir like m6A methyltransferase associated
ZC3H13: Zinc CCCH type
METTL3: Methyltransferase-like protein 3
METTL5: Methyltransferase-like protein 5
METTL14: Methyltransferase-like protein 14
METTL16: Methyltransferase-like protein 16
FTO: obesity-associated protein
ALKBH3: ALKB homolog 3
ALKBH5: ALKB homolog 5
YTH: domain-containing family protein (YTHDF1-3 and YTHDC1-2)
IGF2BP1/2/3: insulin-like growth factor-2 mRNA-binding proteins 1/2/3
HNRNPA2/B1: Heterogeneous nuclear ribonucleoprotein A2/B1
eIF3: eukaryotic translation initiation factor 3
SAM: s-adenosylmethionine
PUFA-PLs: lipid peroxidation of long-chain phospholipid molecules
SLC7A11: solute carrier family 7 member 11
SLC3A2: solute carrier family 3 member 2
GPX4: Glutathione peroxidase 4
ncRNAs: non-coding RNAs
lncRNAs: long non-coding RNAs
miRNAs: microRNAs
pri-miRNAs: Primary microRNAs
mRNAs: message RNAs
circRNAs: Circular RNAs
CDS: coding sequences
NSCLC: non-cellular lung cancer
CRC: colorectal cancer
NPC: nasopharyngeal carcinoma
BC: breast cancer
GC: gastric cancer
HCC: hepatocellular carcinoma
AML: acute myeloid leukemia
GBM: glioblastoma
ESCC: esophageal squamous cell carcinoma
LUAD: lung adenocarcinoma
PDAC: pancreatic ductal adenocarcinoma
HPSCC: hypopharyngeal squamous cell carcinoma
OSCC: oral squamous cell carcinoma
PCa: prostate cancer
MDSCs: myeloid-derived suppressor cells
HB: Hepatoblastoma
CC: Cholangiocarcinoma
PTC: Rapillary thyroid cancer
EMT: epithelial-mesenchymal transition
FZD7: frizzled7
KCNK15-AS1: potassium two pore domain channel subfamily K member 15 and WISP2 antisense RNA 1
TAMs: tumor-associated macrophages
TICs: bladder tumor initiating cells
HER2: human epidermal growth factor receptor 2
FGFR4: fibroblast growth factor receptor 4
GSK3β: glycogen synthase kinase 3β
NFE2L2/NRF2: erythroid-derived 2-like 2
MALAT1: metastasis-associated lung adenocarcinoma transcript 1
MAT2A: methionine adenosyltransferase 2A
GSH/GSSG: the ratio of glutathione to glutathione disulfide
HIF-1α: hypoxia-inducible factor-1α
PABPC1: cytoplasmic poly(A)-binding protein-1
RUNX1-IT1: RUNX1-Intron Transcript 1
LLPS: liquid-liquid phase separation
TKIs: tyrosine kinase inhibitors
m6A1832: 18S rRNA 1832
